# New Cementitious Materials: Advances, Challenges, and Future Perspectives

**DOI:** 10.3390/ma19112243

**Published:** 2026-05-26

**Authors:** Mohammad Saberian

**Affiliations:** School of Engineering, RMIT University, Melbourne, VIC 3000, Australia; mohammad.saberian@rmit.edu.au

## 1. Introduction

The development of cementitious materials has undergone a significant transformation in recent years, driven by the need to reduce the environmental footprint of construction while improving material performance [1,2]. Conventional Portland cement systems, although essential in modern infrastructure, are associated with high CO_2_ emissions and resource consumption [3,4,5]. This has led to a shift toward low-carbon, resource-efficient, and high-performance alternatives [6,7,8].

Recent research has focused on three main directions: first, the valorization of industrial by-products and waste materials, such as biomass ash, steel slag, sludge ash, and recycled aggregates, as supplementary cementitious materials (SCMs) to reduce clinker content and promote the principles of the circular economy; second, the enhancement of material performance through nanomaterials, fibers, and advanced chemical admixtures, which enable improved strength, durability, and functionality [9,10,11,12]; and third, the emergence of additive manufacturing technologies, particularly 3D printing, which introduces new requirements for rheology, buildability, and early-age behavior [13,14,15].

In parallel with these developments, increasing attention has been directed toward understanding the relationships among material composition, hydration mechanisms, microstructural evolution, and long-term engineering performance of advanced cementitious systems [16,17,18]. Studies have shown that the incorporation of alternative binders and functional additives can substantially modify hydration kinetics, pore structure characteristics, interfacial transition zones, and transport properties, thereby influencing mechanical behavior and durability performance [19,20]. Moreover, recent investigations have increasingly integrated experimental observations with numerical modeling and data-driven approaches to optimize material design and predict performance under complex service conditions [21].

Despite these advances, several important challenges remain. Achieving a balance between sustainability and performance is still difficult, particularly when incorporating high volumes of waste-derived materials with variable properties. In addition, the long-term behavior of cementitious materials under coupled environmental conditions is not yet fully understood, limiting reliable service-life predictions. Furthermore, there is a lack of integrated design frameworks linking material composition, microstructure, and macroscopic performance, especially for emerging construction technologies.

The Special Issue entitled “Preparation and Properties of New Cementitious Materials (2nd Edition)” was therefore launched to address these challenges by presenting recent advances in sustainable material design, performance optimization, and innovative construction approaches. The contributions included in this Special Issue provide valuable insights into waste valorization, durability under complex conditions, and materials tailored for next-generation construction technologies. This Special Issue comprises fifteen high-quality papers, including both original research articles and review papers, reflecting the multidisciplinary and rapidly evolving nature of the field.

## 2. Overview of Published Contributions

The contributions in this Special Issue collectively address key themes such as sustainability, material innovation, durability, microstructural behavior, and advanced construction techniques.

The study by Hossain and Hossain (contribution 1) investigated alkali-activated mortars incorporating MgO and nanomaterials, including multi-walled carbon nanotubes and reduced graphene oxide. Their results demonstrated that nano-additives significantly influence rheological behavior, leading to increased viscosity and reduced workability, while also enhancing compressive strength and microstructural densification. The work highlights the potential of combining MgO and nanomaterials to develop high-performance, sustainable alkali-activated systems.

The effects of elevated temperatures and subsequent rehydration on cement paste microstructure and mechanical properties were examined by Xu et al. (contribution 2). Their findings revealed that moderate thermal exposure followed by rehydration can partially restore micro-mechanical properties, whereas excessive temperatures lead to irreversible structural damage. This study provides valuable insights into fire-damaged concrete recovery.

Jiang et al. (contribution 3) explored the performance of ultra-high-performance concrete (UHPC) containing recycled aggregates, basalt fibers, and nanoalumina under high-temperature conditions. The incorporation of fibers and nanomaterials improved crack resistance, thermal stability, and overall durability, demonstrating a promising pathway for sustainable UHPC development.

The use of activated volcanic ash as a high-volume cement replacement was investigated by Játiva et al. (contribution 4). Through thermal and chemical activation, the reactivity of volcanic ash was significantly enhanced, resulting in improved mechanical strength, reduced shrinkage, and increased durability. This study highlights the potential of natural pozzolans in low-carbon cement systems.

Gwóźdź-Lasoń et al. (contribution 5) analyzed the physical properties of foamed concrete incorporating polystyrene granules and synthetic foaming agents. The results showed that increasing polystyrene granule content reduces density and thermal conductivity but also leads to decreased mechanical strength. The study contributes to the development of lightweight, energy-efficient construction materials.

Wang et al. [11] focused on sulfoaluminate cement systems activated with seawater and mineral admixtures. Their findings indicated that optimized combinations of fly ash, silica fume, and slag significantly enhance corrosion resistance and mechanical performance, particularly in marine environments.

Na et al. (contribution 6) investigated asphalt mixtures for railway applications, focusing on mechanical performance and durability. The study demonstrated that modified asphalt binders enhance tensile strength and rutting resistance, supporting their suitability for heavy-load infrastructure applications.

Zafar et al. [15] explored the 3D printability of engineered cementitious composites using internal curing via pumice aggregates. The results showed improved printability, buildability, and sustainability, particularly in water-scarce environments, highlighting the potential of additive manufacturing in construction.

Tram et al. (contribution 7) evaluated the use of bottom ash from municipal waste incineration as a secondary binder. Their findings demonstrated that particle size reduction and thermal treatment significantly improve compressive strength and reactivity, supporting the reuse of waste materials in construction.

The mechanical behavior of traditional and 3D-printed mortar was compared in the study of Gil-Lopez et al. [14]. The research revealed that 3D-printed materials exhibit enhanced flexibility and energy absorption, while conventional mortars provide more predictable stiffness, indicating different application potentials.

Gholizadeh-Vayghan et al. [6] investigated thermally reactivated hydrated cement paste as a supplementary cementitious material. Their results showed that thermal treatment improves reactivity but may affect hydration kinetics and early strength development, highlighting the need for optimized processing conditions.

Lv et al. [12] studied sintered sludge ash as a low-carbon supplementary cementitious material. Through mechanical–thermal activation, significant improvements in mechanical properties were achieved, while life cycle assessment results confirmed reduced environmental impacts.

## 3. Key Insights and Emerging Trends

Several important trends emerge from the contributions in this Special Issue.

First, there is a strong emphasis on sustainability and circular economy principles, with extensive use of industrial by-products such as biomass ash, bottom ash, sludge ash, steel slag, and recycled aggregates. These approaches aim to reduce clinker consumption and promote resource efficiency.

Second, material performance is increasingly evaluated under complex environmental conditions, including high temperatures, marine exposure, and arid climates. This reflects a shift toward designing materials for real-world service conditions rather than ideal laboratory environments.

Third, the integration of nanotechnology and advanced additives plays a critical role in enhancing mechanical properties, durability, and microstructural stability. Nanomaterials, fibers, and chemical admixtures are widely used to tailor material performance.

Fourth, digital construction technologies, particularly 3D printing, are gaining significant attention. Several studies highlight the importance of rheology, buildability, and material design in enabling additive manufacturing applications.

Finally, there is a growing trend toward multi-scale analysis, combining experimental techniques with microstructural characterization to better understand the relationships between composition, structure, and performance.

## 4. Challenges and Future Perspectives

Despite the significant progress demonstrated, several challenges remain. One major challenge is striking a balance between sustainability and performance, particularly when incorporating high volumes of waste materials. While these materials reduce environmental impact, they may compromise early-age strength or durability if not properly optimized. Another critical issue is the limited understanding of long-term performance, especially under coupled environmental conditions such as temperature, moisture, and chemical exposure. Advanced multi-physics modeling and long-term experimental studies are required. In addition, the field lacks unified design frameworks that link material composition, microstructure, and structural performance. Developing predictive and standardized methodologies will be essential for large-scale implementation.

Emerging research directions include the following:

- Low-carbon and carbon-negative cementitious systems;
- Smart and self-healing materials;
- Data-driven and AI-assisted material design;
- Advanced in situ characterization techniques;
- Scalable 3D printing technologies for infrastructure;
